# [Retracted] Synergistic cytotoxic effects of inorganic phosphate and chemotherapeutic drugs on human osteosarcoma cells

**DOI:** 10.3892/or.2026.9160

**Published:** 2026-07-06

**Authors:** Annamaria Spina, Luca Sorvillo, Emilio Chiosi, Antonietta Esposito, Francesca Di Maiolo, Luigi Sapio, Michele Caraglia, Silvio Naviglio

Oncol Rep 29: 1689–1696, 2013; DOI: 10.3892/or.2013.2306

Subsequently to the publication of the above article, a concerned reader drew the Editor's attention to the fact that, regarding the western blots shown in Fig. 3B on p. 1692, the tubulin bands shown for lanes 4 and 5 (showing the ‘Doxo+Pi’ and ‘Taxol’ experiments) were strikingly similar to the tubulin blots shown for lanes 7 and 8 (showing the ‘5-FU’ and ‘5-FU+Pi’ experiments), suggesting possible inappropriate editing of this figure in the paper. It also came to light that the data in question reappeared in a paper published subsequently by the same research group in the journal *Molecules* a couple of years afterwards. Having assessed these data independently in the Editorial Office, the concerns raised by the reader were judged to have been well founded; therefore, the Editor of *Oncology Reports* has decided that the paper should be retracted from the Journal on account of a lack of confidence in the presented data. The authors were asked for an explanation to account for these concerns, but the Editorial Office did not receive a reply. The Editor apologizes to the readership for any inconvenience caused.

